# Social Connection and Epigenetic Aging: Insights from DNA Methylation Clocks

**DOI:** 10.1111/nyas.70402

**Published:** 2026-09-17

**Authors:** Feifei Bu, Daisy Fancourt

**Affiliations:** ^1^ Social Biobehavioural Research Group, Research Department of Behavioural Science and Health Institute of Epidemiology and Health Care University College London London UK

**Keywords:** epigenetic aging, intersectional MAIHDA, loneliness, SLCMA, social connection, social isolation

## Abstract

Social connection is increasingly recognized as a determinant of healthy aging, yet the biological pathways through which it operates remain underexplored. Using data from the Health and Retirement Study (*n* = 2343), this study examined the associations of different dimensions of social connection (social isolation, loneliness, positive and negative relationship quality) with accelerated epigenetic aging measured by second‐generation and third‐generation clocks. Results from doubly robust estimation analyses revealed that baseline social isolation was associated with both the second‐ and third‐generation clocks at 10‐year follow‐up (e.g., GrimAge2: ATE = 0.79, 95% confidence interval [CI] = [0.26, 1.33]), while loneliness was only associated with the third‐generation clocks (e.g., DunedinPACE: ATE = 0.014, 95% CI = [0.001, 0.027]). Although relationship quality showed limited independent associations, co‐occurring social connection deficits were associated with accelerated epigenetic aging, with the fastest epigenetic aging observed among individuals who were socially isolated, lonely, and with low positive and high negative relationship quality (e.g., DunedinPACE: 1.087, 95% CI = [1.066, 1.107] vs. 1.027, 95% CI = [1.019, 1.035]). Further analyses showed a longitudinal accumulation of social isolation was most strongly associated with accelerated epigenetic aging, whereas earlier loneliness exposures appeared to have the strongest associations. These findings highlight the importance of examining social connection both across multiple domains and over time.

## Graphical abstract

This study examined whether social isolation, loneliness, and relationship quality are associated with accelerated epigenetic aging, as measured by multiple epigenetic clocks. We tested if poor social connection across these domains had any cumulative effect on epigenetic aging, and whether these effects accumulated longitudinally over time.

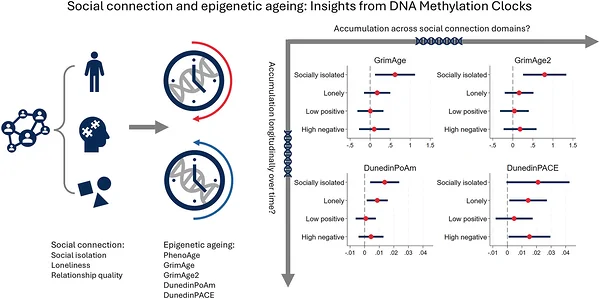

## 1 Introduction

Social connection can be broadly defined as the extent to which an individual is engaged physically, behaviorally, and emotionally with other people [1, 2]. It is a multidimensional construct that includes structural (e.g., social network size, frequency of contact), functional (e.g., social support, loneliness), and quality dimensions [2], with measures comprising both subjective and objective factors [3]. Social connection is fundamental to human wellbeing, influencing psychological, behavioral, and physiological processes [1, 4]. There is growing evidence demonstrating how different aspects of social connection are associated with a variety of health and wellbeing outcomes, such as mortality [5], cardiovascular disease [6], respiratory disease [7], frailty [8], cognitive function [9], depression [10], and so forth.

Many of these studied outcomes relate to processes of aging. And in parallel with such outcomes work, there has been a growing interest in understanding the biological processes underpinning such effects, particularly those that are recognized as biological hallmarks of aging, such as DNA methylation (DNAm). Over the past decade, an increasing number of epigenetic clocks have developed that use DNAm data from various sets of cytosine‐phosphate‐guanine (CpG) sites, genomic loci that are often involved in the regulation of gene expression, to predict chronological age and health‐related outcomes [11]. First‐generation epigenetic clocks were trained to predict chronological age, such as Horvath [12] and Hannum et al. [13]. Second‐generation clocks, for example, PhenoAge [14] and GrimAge [15], were trained to predict mortality and disease morbidity. Most recently, DunedinPoAm and DunedinPACE (arguably the third generation) were trained on longitudinal data and reflect the rate of deterioration in multiorgan systems [16, 17]. By design, these clocks vary in their predictive power of different outcomes, but early studies have shown that second‐generation and, particularly, third‐generation clocks are more sensitive to psychosocial factors than first‐generation clocks [18, 19]. These clocks could provide valuable insights into fundamental molecular processes underpinning the relationship between social connection and age‐related disease susceptibility.

The evidence base for social connection and epigenetic clocks is growing. Recent studies using data from the Health and Retirement Study (HRS, *n* = 1912) suggested that higher levels of social contact and perceived social support (or positive relationship quality), particularly with friends, were associated with decelerated epigenetic aging as measured by GrimAge and DunedinPoAm clocks [18]. In another HRS study (*n* = 3647), perceived social support (or positive relationship quality) across all types (spouse, children, family, friends) was linked to decelerated aging in GrimAge and DunedinPoAm clocks, though there was little evidence on relationship strains (negative relationship quality) [19]. There was also some evidence that having a spouse or child (a measure of social network) was associated with epigenetic aging deceleration [19]. Further evidence from HRS showed that loneliness was associated with GrimAge and DunedinPoAm clocks [20, 21], while social engagement (measured by participation in community activities) was associated with GrimAge [22]. Beyond HRS, Freilich et al. [23] reported that loneliness was associated with Horvath, GrimAge, and DunedinPACE using data from the Midlife in the United States study (*n* = 1310). A Finnish cohort study (*n* = 1539) reported that social satisfaction (measured by perceived social support and satisfaction with life/family) in childhood and adolescence was associated with GrimAge and DunedinPACE clocks in adulthood [24]. A Canadian study (*n* = 1479) found that social participation was associated with decelerated epigenetic aging as measured by the Hannum clock [25]. Another study using Australian data reported cross‐sectional associations of social isolation with GrimAge and DunedinPACE (*n* = 6208), although no evidence was found at 13‐year follow‐up (*n* = 1110) [26]. Existing evidence was largely concerned with second‐ and third‐generation clocks, with only limited and inconsistent evidence on first‐generation clocks, which might reflect differences in study population, social connection measure, and analytical approach.

While these findings advance our understanding of the relationships between social connection and epigenetic aging, critical gaps exist. First, existing studies typically focus on a single aspect of social connection (e.g., social isolation, loneliness, social support, social participation) and have yet to adopt an integrated approach that systematically and simultaneously assesses different dimensions of social connection to assess their relative importance. Second and relatedly, without such an approach, it is unclear whether or to what extent the co‐occurrence or accumulation of different social deficits (e.g., social isolation combined with loneliness) amplifies epigenetic aging acceleration. Third, because most studies rely on baseline exposure assessments, the longitudinal dynamics of social connection are underexplored. Specifically, it is unclear whether timing of social deficits matters and whether there is any long‐term accumulation of deficits over time. Addressing these gaps will deepen our understanding of how different dimensions of social connection become biologically embedded and inform targeted interventions to promote healthy aging.

Using data from HRS, the aims of this study are threefold: (1) to examine whether structural, functional, and quality as distinct dimensions of social connection are associated with epigenetic aging; (2) to assess whether additive (sum of individual effects) or interaction/synergistic effects (combined effects greater than the sum of individual effects) exist across these dimensions; and (3) to assess whether there are cumulative and specificity effects of social deficits over time.

## 2 Materials and Methods

### 2.1 Data

HRS is a nationally representative study of more than 37,000 individuals over the age of 50 and their partners living in the United States [27]. The initial cohort was first interviewed in 1992 and followed up every 2 years, and the sample is replenished with younger cohorts every 6 years. Since 2006, HRS has collected psychosocial and lifestyle data in each biennial wave from a rotating random 50% subsample of participants using the Leave‐Behind Psychosocial and Lifestyle Questionnaire (LBQ), which included a series of questions on social connection [28]. We combined the 2006 and 2008 LBQ data to get a complete cohort (*n* = 14,768).

In 2016, HRS launched the Venous Blood Study, as part of which DNAm assays were done on a subsample of 4104 participants. The sample consisted of all people who participated in the 2016 Healthy Cognitive Aging Project (HCAP) and provided blood samples, plus younger participants for further HCAP assessments and a subsample of non‐HCAP participants. Among the whole sample, 4018 participants (97.8%) passed quality control. For details on blood collection and DNA methylation data, refer to Faul et al. [29]. Among the 4018 participants, 2434 (60.6%) were matched to the 2006/2008 LBQ data. After excluding participants with missing data (3.7%), we had an analytical sample of 2343 participants (see Figure S1).

### 2.2 Measures

#### 2.2.1 Epigenetic Clocks

HRS initially provided 13 epigenetic clocks developed from the DNAm data. Of these, we excluded all first‐generation clocks as they were primarily trained to predict chronological age without incorporating clinical biomarkers, focusing only on second‐ and third‐generation clocks: PhenoAge, GrimAge, and DunedinPoAm clocks. In 2025, HRS released two additional newer clocks, namely, GrimAge2 and DunedinPACE, which represent updated versions of their predecessors, respectively. We included both versions of each clock to capture recent methodological improvements while allowing for cross‐referencing with prior studies.

PhenoAge was developed to predict mortality risk based on nine markers of tissue and immune function, and chronological age [14]. It is expressed in units of years.

GrimAge was developed based on seven DNAm‐based surrogate markers of plasma proteins and smoking pack‐years regressed on time‐to‐death [15]. GrimAge2 included two additional biomarkers: high‐sensitivity C‐reactive protein and hemoglobin A1C, and was shown to outperform GrimAge in predicting mortality and certain age‐related conditions [30]. Both of these clocks are measured in units of years.

DunedinPoAm was developed based on longitudinal change over time in 18 biomarkers in the cardiovascular, metabolic, renal, hepatic, pulmonary, periodontal, and immune systems [16]. DunedinPACE was an updated version of DunedinPoAm with longer follow‐ups and more reliable DNAm probes [17]. Both of these clocks were measured in rates of biological aging per chronological year, with values greater than 1 indicating accelerated aging.

#### 2.2.2 Social Connection

Structural dimension: Social isolation was adapted from the Steptoe social isolation index [31]—an objective measure of social connection—by assigning 1 point to each of the following factors: (1) unmarried; (2) less than monthly contact (face‐to‐face, telephone, or writing/email) with children; (3) less than monthly contact (face‐to‐face, telephone, or writing/email) with other family; (4) less than monthly contact (face‐to‐face, telephone, or writing/email) with friends; (5) less than monthly attendance at nonreligious meetings or programs of groups, clubs, or organizations. The total score ranged from 0 to 5. Participants with a score of 3 or more were defined as being socially isolated (coded 1) versus not isolated (coded 0).

Functional dimension: Loneliness was measured using the three‐item UCLA loneliness scale (Cronbach's alpha 0.72 [32]), which assesses subjective perceptions of deficits in social connection. The questions include: (1) how often do you feel lack companionship? (2) how often do you feel isolated from others? (3) how often do you feel left out? Responses to each question were scored on a 3‐point Likert scale ranging from hardly ever/never, to some of the time, to often. Using the sum score, we had a loneliness scale ranging from 3 to 9, with a higher score indicating a higher level of loneliness. Participants with a score of 6 or more were defined as lonely (coded 1) versus not lonely (coded 0) [33].

Quality dimension: Relationship quality was measured using four sets of questions on participants’ subjective reporting of their relationship with their spouse, children, other family, and friends, respectively. These included three items on positive quality (Cronbach's alpha 0.81−0.86 across relationship categories [28]): (1) how much do they really understand the way you feel about things? (2) how much can you rely on them if you have a serious problem? (3) how much can you open up to them if you need to talk about your worries? Another four items were on negative quality (Cronbach's alpha 0.76−0.79 across relationship categories [28]): (1) how often do they make too many demands on you? (2) how much do they criticize you? (3) how much do they let you down when you are counting on them? (4) how much do they get on your nerves? Each item was measured on a 4‐point Likert scale (a lot, some, a little, not at all). The positive and negative scales were calculated separately by taking averages across spouse, children, other family, and friends. All items were reversed, so a higher score indicates better quality on the positive scale, and poor quality on the negative scale. These scales were then dichotomized, with low positive quality defined as the lowest quartile (coded 1) versus all others (coded 0), and high negative quality defined as the highest quartile (coded 1) versus all others (coded 0).

### 2.3 Covariates

We considered a range of demographic and socioeconomic covariates including age, age‐squared, sex (female, male), race/ethnicity (white/Caucasian, black/African American, other), education (no qualification, high school/GED, college, postgraduate), household income quartiles, and employment status (employed vs. other). We also accounted for health‐related factors, including smoking (never smoker, ex‐smoker, current smoker), diagnosed psychiatric conditions (yes, no), and diagnosed physical health conditions (yes, no). It is possible that the health‐related factors are on the causal pathway between social connection and epigenetic aging, such that controlling for them may underestimate the total association. However, smoking is well‐established as the strongest behavioral predictor of epigenetic aging [34], and health conditions are likely to be confounders that influence both social connection and epigenetic aging. Therefore, the risk of overadjustment was considered to be outweighed by potential biases of omitting these factors. All covariates listed above were derived from baseline. Finally, we controlled for the year of baseline data collection (2006, 2008) to account for the discrepancy in follow‐up period and cell types from the DNAm data (natural killer cell, B cell, CD4 T cell, CD8 T cell, and monocyte).

### 2.4 Statistical Analysis

For aim 1, we applied doubly robust estimation using inverse‐probability‐weighted regression adjustment (IPWRA), fitting separate models for each social connection measure at baseline (exposure) and each epigenetic clock (outcome). This method involves building two models to account for the nonrandom treatment assignment: (i) a regression adjustment model for the outcome; (ii) a treatment‐assignment model for the exposure (see Supplementary Methods for algebraic specification). It uses weighted regression coefficients to compute averages of treatment‐level predicted outcomes, where the weights are the estimated inverse probabilities obtained from the treatment‐assignment model. This analytical approach has the double‐robust property: it only requires that either the outcome or treatment‐assignment model is correctly specified, not both. Both models controlled for the sociodemographic and health‐related covariates, and baseline year. The outcome model additionally controlled for cell type variables.

For aim 2, we conducted intersectional multilevel analysis of individual heterogeneity and discriminatory accuracy (MAIHDA) analysis to examine how different social connection dimensions intersect to influence biological aging, allowing for both additive and interaction/synergistic effects [35]. It involved creating strata by combining different social connection measures at baseline (socially isolated, lonely, positive and negative quality), forming a multilevel data structure, with individuals nested in strata. The 16 possible strata had observed sizes ranging from 13 to 1101. The dichotomization of exposure was primarily to ensure decent strata size for intersectional MAIHDA. Data were analyzed using standard multilevel modeling, starting from a null model without any explanatory variables to quantify total variance between strata. Subsequently, additive main effects models were fitted by including social connection measures as fixed level‐two variables. Potential interaction effects between social connection measures were captured by a stratum random effect (see Supplementary Methods for algebraic specification). The MAIHDA model was fitted separately for different epigenetic clocks.

For aim 3, we used the structured life course modeling approach (SLCMA) to test associations of epigenetic aging with timing (sensitive periods: early, middle, late waves) and accumulation (sum across waves) of social connection over time. SLCMA is a two‐stage approach [36]. In the first stage, it uses least‐angle regression to test a series of single and compound hypotheses, identifying the hypothesis with the greatest explanatory power. This is typically determined by elbow plots of variance explained. In the second stage, it calculates effect estimates, *p* values, and confidence intervals of the selected model in the first stage (see Supplementary Methods for algebraic specification). Unlike aim 1 and 2 analyses, the SLCMA models used repeated measures of social connection across three waves between 2006 and 2016, and were fitted separately for each social connection measure and epigenetic clock.

To test robustness of results, sensitivity analyses were conducted excluding health‐related covariates that could be on the causal pathway. For the doubly robust estimation model and intersectional MAIHDA, complete case analyses were used given missing data were minimal (3.7%). For SLCMA, missing data in time‐varying social connection measures were imputed using multiple imputation by chained equations under the assumption of missing‐at‐random. Twenty imputed datasets were generated, with the imputation model including social connection and epigenetic measures, as well as all covariates used in the main analyses (see Table S1 comparing participants with complete and incomplete data). All analyses were conducted in Stata V18, except for SLCMA, which was fitted in R 4.4.1, using the *slcma* package [37].

## 3 Results

The average age of our analytical sample was 64.92, with a standard deviation (SD) of 8.44 (Table 1). About 40% of participants were males, 84.8% were white/Caucasian, 10.4% were black/African American, and 4.8% were from other ethnic minority backgrounds. In the sample, 13.3% of participants were socially isolated, and 25.9% reported being lonely, 23.5% in the low positive and 23.4% in the high negative relationship quality groups. The analytical sample was broadly comparable to the overall LBQ cohort in most characteristics, but demonstrated a younger age distribution, higher percentage of employment, and slightly lower percentage of social isolation (Table 1). These observed discrepancies, to some extent, likely reflected the DNAm sample being a relatively younger birth cohort.

**TABLE 1:** Analytical sample characteristics compared to the original LBQ (2006/2008) and DNAm (2016) datasets: Mean (SD)/%.

Variables		Analytical sample (*N* = 2343)	LBQ 2006/2008 (*N* = 14,768)	DNAm data 2016 (*N* = 4018)
Age	Age	64.92 (8.44)	68.34 (10.53)	60.44 (9.68)^†^
Gender	Female	60.22%	59.32%	58.46%
	Male	39.78%	40.68%	41.54%
Ethnicity	White/Caucasian	84.81%	82.58%	75.02%
	Black/African American	10.37%	12.97%	16.78%
	Other	4.82%	4.45%	8.19%
Education	None	15.32%	19.98%	16.96%
	High school/GED	54.97%	54.46%	52.63%
	College	19.16%	16.76%	20.68%
	Postgraduate	10.54%	8.80%	9.73%
Household income	Q 1 (lowest)	16.13%	23.44%	22.47%
	Q 2	24.93%	27.51%	26.21%
	Q 3	30.26%	26.33%	26.18%
	Q 4 (highest)	28.68%	22.72%	25.14%
Employment status	Employed	42.34%	31.45%	29.17%
	Other	57.66%	68.55%	70.83%
Diagnosed psychiatric condition	Yes	20.61%	20.99%	25.54%
	No	79.39%	79.01%	74.46%
Diagnosed physical health condition	Yes	83.01%	85.69%	90.37%
	No	16.99%	14.31%	9.63%
Smoking status	Never smoker	44.52%	43.19%	44.17%
	Ex‐smoker	43.19%	43.56%	44.44%
	Current smoker	12.29%	13.25%	11.39%
Socially isolated	Yes	13.32%	16.18%	—
	No	86.68%	83.82%	—
Lonely	Yes	25.91%	27.03%	—
	No	74.09%	72.97%	—
Low positive quality	Yes	23.47%	23.18%	—
	No	76.53%	76.82%	—
High negative quality	Yes	23.43%	23.38%	—
	No	76.57%	76.62%	—

† Age for the DNAm sample was adjusted in 2006/2008, but other covariates were measured in 2016.

### 3.1 Aim 1

**FIGURE 1:**
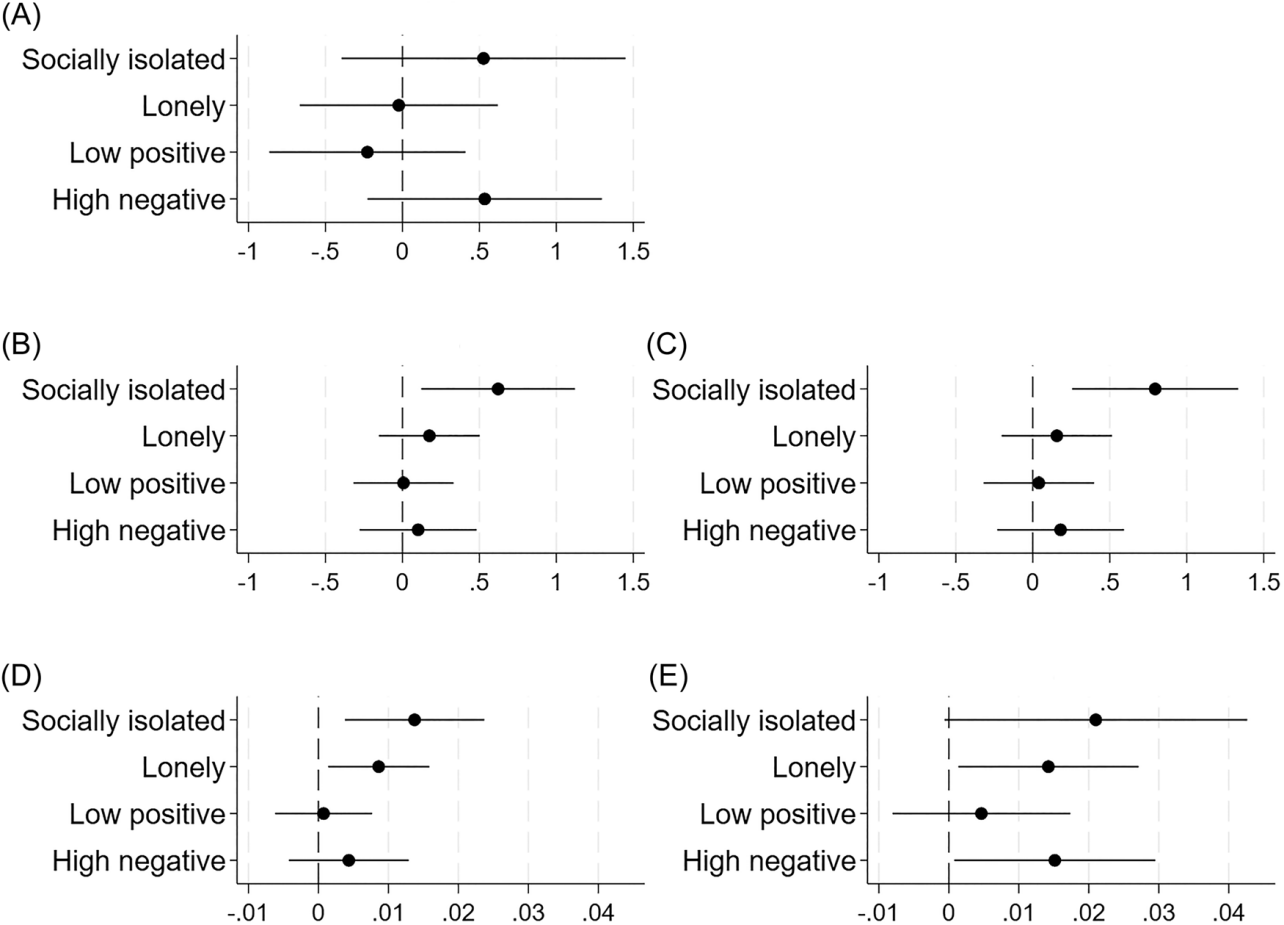
Estimated ATE and 95% CIs from the doubly robust estimation models on (A) PhenoAge, (B) GrimAge, (C) GrimAge2, (D) DunedinPoAm, and (E) DunedinPACE.

Results from the doubly robust estimation models are reported in Figure 1 (see also Table S2). Social isolation was consistently associated with epigenetic aging acceleration for most of the clocks, except for PhenoAge. Specifically, participants who were socially isolated had an epigenetic age of 0.62 years older on GrimAge compared to those who were not isolated (95% CI = [0.12, 1.12], *p* = 0.004), and 0.79 years older on GrimAge2 (95% CI = [0.26, 1.33], *p* = 0.004). Social isolation was associated with a faster pace of aging by 0.014 years for every year of chronological age for DunedinPoAm (95% CI = [0.004, 0.024], *p* = 0.007), and by 0.021 for DunedinPACE, which was only marginally significant (95% CI = [−0.001, 0.043], *p* = 0.057).

There was no evidence that loneliness was associated with PhenoAge, GrimAge, or GrimAge2. However, participants who were lonely aged at a faster pace by 0.009 years for every year of chronological age for DunedinPoAm (95% CI = [0.001, 0.016], *p* = 0.019), and by 0.014 for DunedinPACE (95% CI = [0.001, 0.027], *p* = 0.030). There was no evidence that positive or negative relationship quality was associated with any of the clocks, except that negative quality was associated with a faster pace of aging by 0.015 for DunedinPACE (95% CI = [0.001, 0.029], *p* = 0.039).

Results from sensitivity analyses excluding health‐related covariates, which may theoretically lie on the causal pathway, were consistent with the main findings, but effect sizes were larger as anticipated (Table S3).

### 3.2 Aim 2

Table 2 shows the results from the intersectional MAIDHA analyses focusing on the third‐generation clocks (DunedinPoAm and DunedinPACE), which showed stronger evidence of association with social connection measures. Using DunedinPoAm as the example, from the null model, we see that only 1.26% of total variance lay between social connection strata, indicating a relatively small amount of clustering at the stratum level (model 1). After adding social connection measures in model 2, the variance partition coefficient was nearly zero, suggesting there is almost no between‐stratum variance left unexplained. This was consistent with having a proportional change in variance of 100%, and all stratum random effects were close to zero. In other words, all between‐stratum variance in the null model was explained by additive main effects, so there was no evidence for interaction effects.

**TABLE 2:** Results from the intersectional MAIHDA analyses (*N* = 2343).

		Model 1			Model 2	
	Coef.	95% CI	*p*	Coef.	95% CI	*p*
DunedinPoAm						
**Fixed effects**						
Socially isolated (yes vs. no)				0.017	[0.006, 0.028]	0.002
Lonely (yes vs. no)				0.010	[0.001, 0.018]	0.032
Low positive quality (yes vs. no)				0.004	[−0.004, 0.013]	0.325
High negative quality (yes vs. no)				0.007	[−0.002, 0.016]	0.122
Intercept	1.082	[1.075, 1.089]	<0.001	1.067	[1.062, 1.072]	<0.001
**Random effects: variance**						
Stratum‐level ($\sigma_{u}^{2}$)	0.000	[0.000, 0.000]		0.000	[0.000, 0.002]	
Individual‐level ($\sigma_{e}^{2}$)	0.008	[0.007, 0.008]		0.008	[0.007, 0.008]	
**Summary statistics**						
Variance partition coefficient (VPC)	1.26%	[0.32%, 4.79%]		0.00%	[0.00%, 0.00%]	
Proportional change in variance (PCV)				100%		
DunedinPACE						
**Fixed effects**						
Socially isolated (yes vs. no)				0.022	[0.004, 0.040]	0.015
Lonely (yes vs. no)				0.020	[0.006, 0.034]	0.006
Low positive quality (yes vs. no)				0.003	[−0.012, 0.017]	0.715
High negative quality (yes vs. no)				0.015	[0.000, 0.029]	0.044
Intercept	1.049	[1.038, 1.059]	<0.001	1.027	[1.019, 1.035]	<0.001
**Random effects: variance**						
Stratum‐level ($\sigma_{u}^{2}$)	0.000	[0.000, 0.000]		0.000	[0.000, 0.000]	
Individual‐level ($\sigma_{e}^{2}$)	0.021	[0.020, 0.022]		0.021	[0.020, 0.022]	
**Summary statistics**						
Variance partition coefficient (VPC)	0.86%	[0.19%, 3.75%]		0.00%	[0.00%, 0.00%]	
Proportional change in variance (PCV)				100%		

**FIGURE 2:**
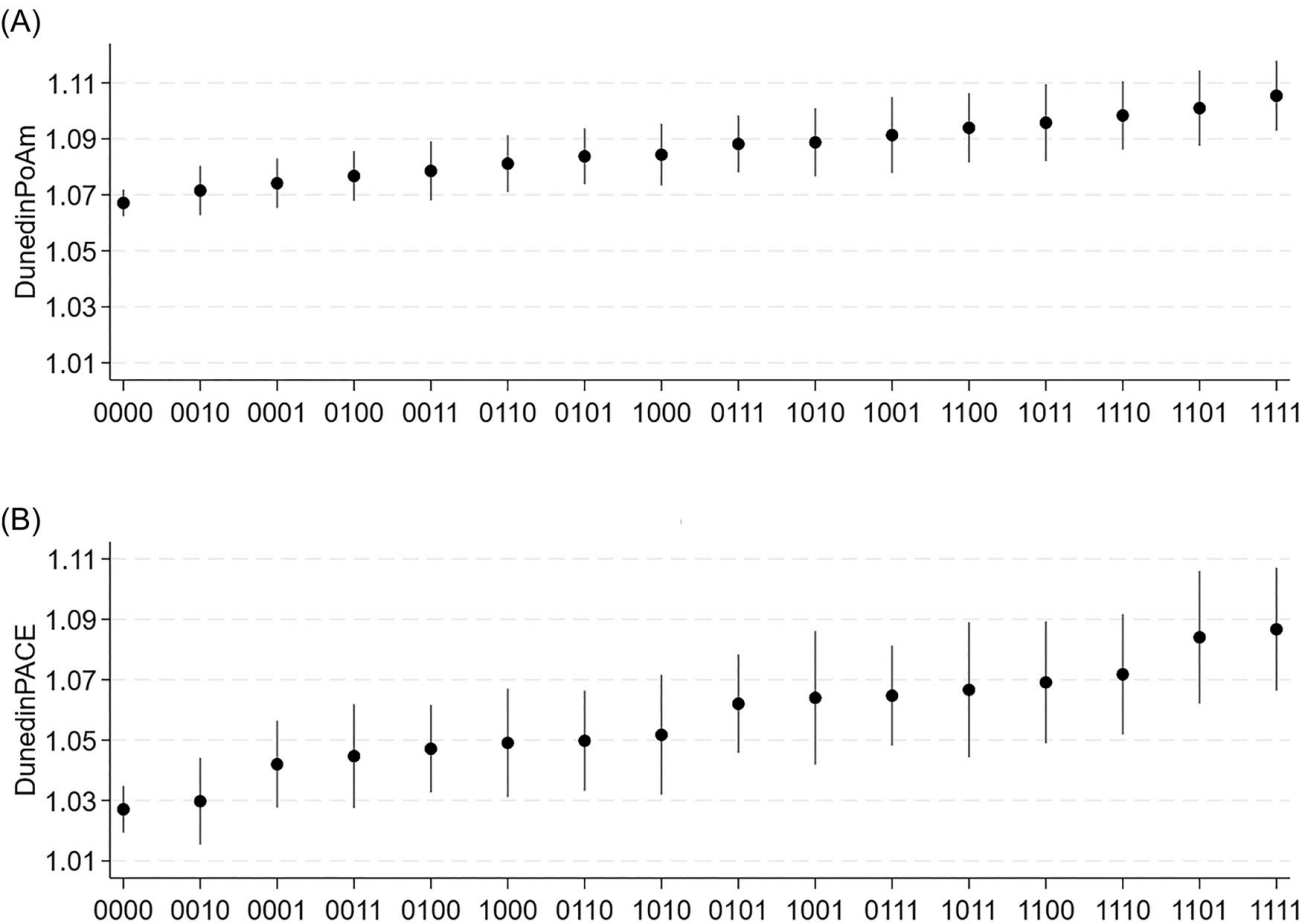
Predicted DunedinPoAm and their 95% CIs by social connection strata from the intersectional MAIDHA on (A) DunedinPoAm and (B) DunedinPACE (strata ID code: socially isolated [0/1], lonely [0/1], low positive quality [0/1], high negative quality [0/1]).

Figure 2A shows the predicted values and their 95% confidence intervals (CI) for each stratum based on model 2. The stratum with the fastest aging rate was consisted of people who were socially isolated, lonely, with low positive and high negative relationship quality (stratum ID: 1111). While some social connection variables were not statistically significant, their cumulative effects might still yield meaningful impacts. For example, the difference between the least and most disadvantaged strata (stratum ID: 0000 vs. 1111) was around 0.04 years for every year of chronological age (1.105, 95% CI = [1.093, 1.118] vs. 1.067, 95% CI = [1.062, 1.072]). The analyses of DunedinPACE yielded similar results, showing an even larger difference: the most disadvantaged strata aged at a faster rate by 0.06 than the least disadvantaged (1.087, 95% CI = [1.066, 1.107] vs. 1.027, 95% CI = [1.019, 1.035], Figure 2B).

### 3.3 Aim 3

**FIGURE 3:**
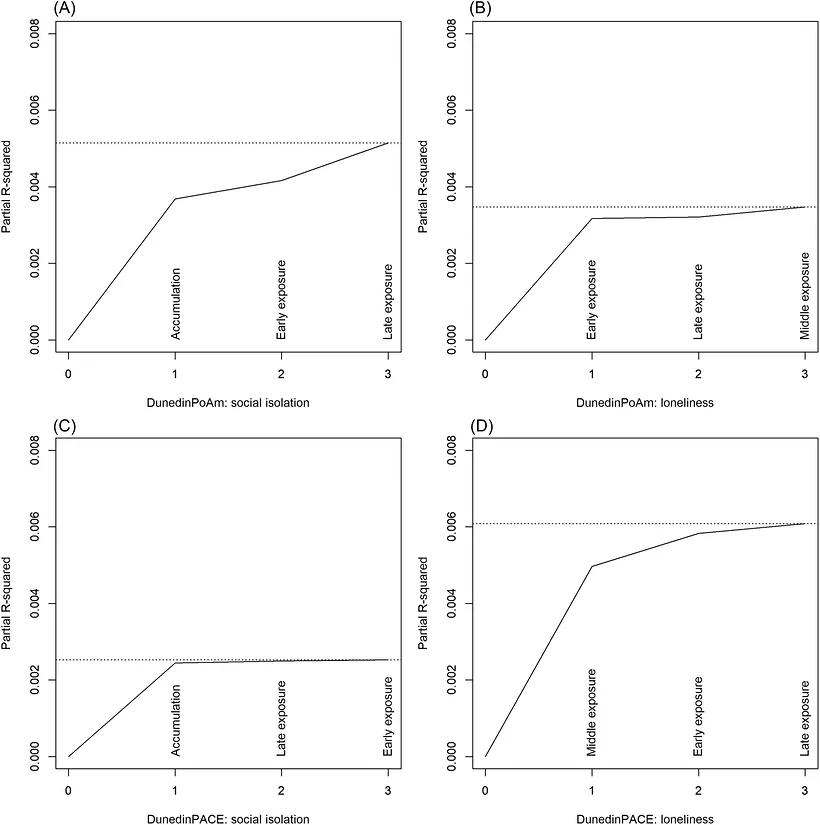
Elbow plots of SLCMA models testing the association of DunedinPoAm and DunedinPACE with social isolation and loneliness.

Our investigation of longitudinal dynamics of social connection focused on social isolation and loneliness as they demonstrated more consistent evidence of association with the third‐generation clocks. Both the DunedinPoAm and DunedinPACE models supported an accumulation model for social isolation (Figure 3A,C), suggesting that social isolation across multiple time points contributed to accelerated epigenetic aging. For loneliness, however, the early‐exposure model was selected for DunedinPoAm and middle‐exposure for DunedinPACE. The results of the selected models are shown in Table S4. Sensitivity analyses excluding health‐related covariates yielded similar results (Figure S2 and Table S5).

## 4 Discussion

Using data from a large nationally representative cohort study of US adults over the age of 50, we found that baseline social isolation was associated with both the second‐ and third‐generation clocks at the end of 10‐year follow‐up, while loneliness was only associated with the third‐generation clocks. There was limited evidence that positive nor negative relationship quality was associated with epigenetic clocks. Intersectional‐MAIHDA analyses supported an additive model, with the fastest epigenetic aging observed among individuals who were socially isolated, lonely, with low positive and high negative relationship quality. The SLCMA models indicated that cumulative social isolation over time is most strongly associated with accelerated aging, while for loneliness, earlier exposure may have the greatest impact.

Our findings on social isolation and loneliness generally align with previous research in demonstrating that deficits in social connection are biologically embedded, as evident through analyses of DNAm patterns in epigenetic clocks [18, 19, 23] and epigenome‐wide association studies [38]. Within our study, social isolation exhibited greatest consistency in its associations with epigenetic aging across different clocks, as well as across different analytical approaches. This accords with the fact that social isolation, compared to loneliness, is more robustly linked to other molecular biomarkers downstream of DNAm, such as plasma proteins, and broader biomarkers of inflammation and cellular dysfunction in studies of human participants [39, 40]. It is also supported by consistent evidence from experimental studies of the biological impacts of social isolation in nonhuman social species (e.g., rodents) [41]. Consistent with a prior study [38], loneliness was associated only with the third‐generation clocks. A plausible explanation is that these clocks are trained on longitudinal changes in clinical biomarkers reflecting the integrity of multiple organ systems. This makes them more likely to capture the biological embedding of loneliness across the genome, as indicated by epigenome‐wide association findings [38]. Moreover, their longitudinal design is better positioned to select DNAm markers that capture causal aging processes rather than those that are simply cross‑sectional correlates.

The overall absence of association between relationship quality and epigenetic aging also aligns with previous findings [19]. However, unlike earlier studies [18, 19], we found no evidence that positive relationship quality (perceived social support) was associated with either GrimAge or DunedinPoAm, despite adjusting for overlapping (though not identical) covariates. A key methodological distinction may explain this discrepancy. While previous studies examined relationship types (spouse, children, family, friends) individually, our study aggregates relationship quality across all types. Notably, Hillmann et al. [18] identified associations only for friend‐specific support, but not other types. It is possible that aggregated measures may obscure type‐specific effects. Further, there is also a methodological difference in measurement timings between studies. Similar to Hillmann et al. [18], our study derived baseline social connection and covariates from the 2006/2008 waves, whereas Rentscher et al. [19] mainly used measures collected in 2014/2016. This may introduce temporal confounding, as the outcome measures (epigenetic clocks) assessed in 2016 may affect people's subjective evaluation of their relationship quality. Future research is needed to investigate this further.

While measures of relationship quality did not reach statistical significance, their co‐occurrence with other social connection measures can have marked impacts on epigenetic aging. Our analyses have shown that the difference between the groups with the most and fewest social deficits is 4% acceleration on DunedinPoAm (6% for DunedinPACE). Although the effect size appears to be small, it is broadly comparable to the estimates reported in other studies of the potential impact of other health‐related factors [42]. While these comparisons are indirect rather than directly assessed in this study, so caution is required in interpreting them (due to variations in methods, data, and confounders adjusted for), they nonetheless imply a meaningful difference [34]. The additive effects of social connection deficits are also consistent with previous studies based on cluster analysis showing that social connection clusters with deficits in any of the structural, functional, and quality dimensions are associated with worse phenotype health indicators [43]. This suggests that studies focusing on single‐exposure effects may underestimate the true burden of social connection deficits. Future studies should adopt a more holistic approach assessing multiple domains to better capture the full extent of the impact of social connection.

Notably, our analyses have shown that nearly all outcome variance is explained by additive effects of social isolation, loneliness, positive and negative relationship qualities, with no evidence of interaction/synergistic effects (combined effects exceeding the sum of individual effects). However, this absence of interaction may be specific to the biological aging process captured by epigenetic clocks assessed in the present study, which primarily reflect pathways related to immune responses, inflammation, and metabolic dysregulation. It is important to note that social connection can influence health outcomes through other biological pathways, or via psychological and behavioral mechanisms, where interaction/synergistic effects may emerge. This underscores the need for future research to explore additive versus interaction/synergistic effects across diverse health and aging domains.

The detrimental effects of social connection deficits not only accumulate across domains but also over time, particularly for social isolation. Loneliness, however, appears more time‐sensitive, demonstrating its strongest associations with epigenetic aging when experienced at an earlier time point. This difference may stem from their distinct biological embedding processes. Social isolation, as a chronic stressor, has been more robustly linked to systemic inflammation (e.g., C‐reactive protein, fibrinogen) [40] and cellular dysfunction [39], which are likely to compound through prolonged exposure. In contrast, loneliness has a more indirect effect through the activation of neuroendocrine stress pathways (e.g., hypothalamic pituitary adrenocortical axis) [40], which may have threshold effects. For instance, once cortisol regulation is impaired, additional loneliness may not further accelerate epigenetic aging. However, the absence of observed accumulation effects for loneliness should be interpreted with caution. First, there may be a delayed biological embedding for loneliness, or the study's follow‐up period may not be long enough to capture the impact of loneliness in recent years with respect to the epigenetic measures. Second, loneliness as a self‐reported subjective measure is more prone to measurement noise, making it less sensitive to detect longitudinal accumulation effects. Finally, HRS captures data for adults aged 50 and above, so we were unable to explore how earlier exposure in the life‐course influences epigenetic aging.

Our study has many strengths, including using data from a nationally representative study, a holistic consideration of different social connection aspects (subjective, objective) and dimensions (structural, functional, quality), and investigation of cross‐sectional and longitudinal exposure patterns using a triangulation of statistical approaches. However, there are some limitations. First, our analytical sample is only a fraction of the HRS sample. Although the sample characteristics are largely in line with the original sample, some differences do exist, such as age and employment status. Therefore, the generalizability of the findings could be compromised to some extent. Second, we controlled for a range of confounding factors identified using directed acyclic graphs informed by the existing literature. However, we cannot rule out the possibility of unmeasured confounding biases. Third, we acknowledge that epigenetic clocks are high‐level summary measures that do not directly reveal the specific molecular mechanisms underlying social connection. Future research employing epigenome‐wide association analysis to pinpoint individual CpG sites linked to different aspects of social connection would provide valuable mechanistic insights.

## 5 Conclusion

Overall, our results provide empirical evidence that social isolation and loneliness is related to epigenetic aging, particularly when isolation accumulates across older adulthood or when loneliness is experienced earlier. Although the impact of relationship quality appears to be small and statistically insignificant, it may compound with social isolation and loneliness in an additive way to drive a practically meaningful impact. Our study underscores the importance of assessing social connection holistically across different domains, and longitudinally over time.

## Author Contributions

F.B. and D.F. conceived the study. F.B. analyzed the data. F.B. and D.F. wrote the first draft. Both authors provided critical revisions. Both authors read and approved the submitted manuscript.

## Conflicts of Interest

The authors declare no conflicts of interest.

## Supporting information




**Supporting File 1**: nyas70402‐sup‐0001‐SuppMat.docx
