# Supplementary material for: Social Connection and Epigenetic Aging: Insights from DNA Methylation Clocks
Source: Ann N Y Acad Sci. 2026 Sep 17;1563(1):e70402. doi: 10.1111/nyas.70402 (PMC13585062; doi:10.1111/nyas.70402)
Supplement: Supplementary file 1 [file NYAS-1563-0-s001.docx]

Social connection and epigenetic ageing: Insights from DNA Methylation Clocks

Supplementary Materials

Table of Contents

[Supplementary Methods: Algebraic specification of the statistical models	2](#_Toc239160295)

[1. Double robust estimation using inverse-probability-weighted regression adjustment IPWRA	2](#_Toc239160296)

[2. Intersectional multilevel analysis of individual heterogeneity and discriminatory accuracy (MAIHDA)	2](#_Toc239160297)

[3. Structured life course modelling approach (SLCMA)	3](#_Toc239160298)

[Table S1. Comparing baseline characteristics of participants with complete and incomplete data in any of the social connection measures (social isolation, loneliness, positive and negative relationship quality) in SLCMA analysis (N=2313)	4](#_Toc239160299)

[Table S2. Results from doubly robust estimation models (N=2343)	5](#_Toc239160300)

[Table S3. Results from doubly robust estimation models: sensitivity analysis excluding health-related covariates (N=2343)	5](#_Toc239160301)

[Tables S4. Results from SLCMA models on DunedinPoAm and DunedinPACE: social isolation and loneliness (N=2313†)	6](#_Toc239160302)

[Tables S5. Results from SLCMA models on DunedinPoAm and DunedinPACE sensitivity analysis excluding health-related covariates: social isolation and loneliness (N=2313†)	6](#_Toc239160303)

[Figure S1. Sample selection diagram	7](#_Toc239160304)

[Figure S2. Elbow plots of SLCMA models testing the association of DunedinPoAm and DunedinPACE with social isolation and loneliness: sensitivity analysis excluding health-related covariates	8](#_Toc239160305)

# Supplementary Methods: Algebraic specification of the statistical models

## 1. Double robust estimation using inverse-probability-weighted regression adjustment IPWRA

Let $Y_{i}$ denote the continuous outcome for individual *i*, $D_{i}$ the binary exposure, and $X_{i}$ a vector of observed covariates.

The conditional probability of exposure was estimated as:

$$p_{i}=P\left( D_{i}=1 \right| X_{i})= g^{-1} (\alpha_{0}+ X_{i}^{T}\alpha)$$

where $g^{-1}(.)$ is the inverse-link function.

The exposure-specific inverse-probability weights are:

$$w_{i}= \left\{ \begin{aligned} \frac{1}{p_{i}} if D_{i}=1 \\ \frac{1}{1-p_{i}} if D_{i}=0 \end{aligned} \right.$$

The outcome model is:

$$E\left( Y_{i} \right|D_{i}=d, X_{i})= \beta_{0d}+ X_{i}^{T}\beta_{d}, d \epsilon\left\{ 0, 1 \right\}$$

For each treatment group $d \epsilon\left\{ 0, 1 \right\}$, the coefficients are estimated as:

$$\hat{\beta}_{d}=\begin{matrix} \arg min \\ \beta_{d} \end{matrix}\sum_{i:D_{i}=d} w_{i}{(Y_{i}-\beta_{0d}-X_{i}^{T}\beta_{d})}^{2}$$

The average treatment effect is estimated as:

$$\hat{ATE}= \frac{1}{N}\sum_{i=1}^{N} \left[ \hat{Y_{i}}\left( 1 \right)- \hat{Y_{i}}\left( 0 \right) \right]$$

## 2. Intersectional multilevel analysis of individual heterogeneity and discriminatory accuracy (MAIHDA)

Let $Y_{ij}$ denotes the continuous outcome for individual *i* in stratum *j,* $u_{j}$is the stratum random effects, $e_{ij}$the residual term for individual *i* in stratum *j.*

The null model is:

$$Y_{it}= \beta_{0}+ u_{j}+ \varepsilon_{ij}$$

$$u_{j}\sim N\left( 0, \sigma_{u}^{2} \right)$$

$$\varepsilon_{ij}\sim N\left( 0, \sigma_{\varepsilon}^{2} \right)$$

$$VPC= \frac{\sigma_{u}^{2}}{\sigma_{u}^{2}+ \sigma_{\varepsilon}^{2}}$$

The additive main-effects model is:

$$Y_{it}= \beta_{0}+\beta_{1}X_{1j}+\ldots+ \beta_{k}X_{1k}+ u_{j}+ \varepsilon_{ij}$$

$$u_{j}\sim N\left( 0, \sigma_{u}^{2} \right)$$

$$\varepsilon_{ij}\sim N\left( 0, \sigma_{\varepsilon}^{2} \right)$$

Please refer to Evans et al. (2024) for further details.

## 3. Structured life course modelling approach (SLCMA)

Let $Y_{i}$ denote the continuous outcome for individual *i*, $X_{it}$ the exposure for individual i at time t, $C_{i}$ a vector of observed covariates, $e_{i}$ the residual term

Each life-course hypothesis was represented by an encoded variable, denoted by $H_{hi}$, where $h=1, \ldots, H$ identifies the candidate hypothesis. The general model was:

$$Y_{i}=\alpha+ \sum_{h=1}^{H} \beta_{h}H_{hi}+\gamma^{T}C_{i}+\varepsilon_{i}$$

$$\varepsilon_{i}\sim N(0, \sigma^{2})$$

A sensitive-period hypothesis proposes that exposure at a particular measurement occasion is more strongly associated with the later outcome than exposure at other occasions, where sensitive-period variable is defined as:

$$S_{it}=X_{it}$$

The accumulation hypothesis proposes that the association with the outcome increases with the number of occasions on which an individual is exposed, regardless of when those exposures occur.

The accumulation score was calculated as:

$$A_{i}=\sum_{t=1}^{T} X_{it}$$

The recency hypothesis proposes that more recent exposure is more strongly associated with the outcome than exposure occurring further in the past. A recency score was constructed as a weighted sum:

$$R_{i}=\sum_{t=1}^{T} {w_{t}X}_{it}$$

$$w_{1}<w_{2}<\ldots<w_{T}$$

Please refer to Zhu et al. (2021) and Simth (2024) for further details.

# Table S1. Comparing baseline characteristics of participants with complete and incomplete data in any of the social connection measures (social isolation, loneliness, positive and negative relationship quality) in SLCMA analysis (N=2313)

| Variables |  | Complete  (N=1864) | Incomplete (N=449) | p^†^ |
| --- | --- | --- | --- | --- |
| Age | Age | 64.94 (8.31) | 64.73 (8.98) | 0.634 |
| Gender | Female | 60.30% | 60.36% | 0.983 |
|  | Male | 39.70% | 39.64% |  |
| Ethnicity | White/Caucasian | 86.53% | 77.51% | <0.001 |
|  | Black/African American | 9.12% | 15.37% |  |
|  | Other | 4.35% | 7.13% |  |
| Education | None | 11.75% | 29.84% | <0.001 |
|  | High School/GED | 57.14% | 45.43% |  |
|  | College | 19.90% | 16.48% |  |
|  | Postgraduate | 11.21% | 8.24% |  |
| Household income | Q 1 (lowest) | 14.16% | 24.05% | <0.001 |
|  | Q 2 | 24.62% | 26.06% |  |
|  | Q 3 | 31.28% | 26.50% |  |
|  | Q 4 (highest) | 29.94% | 23.39% |  |
| Employment status | Employed | 43.29% | 38.98% | 0.097 |
|  | Other | 56.71% | 61.02% |  |
| Diagnosed psychiatric condition | Yes | 18.94% | 27.39% | <0.001 |
|  | No | 81.06% | 72.61% |  |
| Diagnosed physical health condition | Yes | 82.51% | 84.86% | 0.235 |
|  | No | 17.49% | 15.14% |  |
| Smoking status | Never smoker | 45.17% | 42.76% | 0.005 |
|  | Ex-smoker | 43.72% | 40.53% |  |
|  | Current smoker | 11.11% | 16.70% |  |

Note: † p values are from independent t-test for age, and chi-squared test for all other variables

# Table S2. Results from doubly robust estimation models (N=2343)

|  |  | PhenoAge |  |  | GrimAge |  |  | GrimAge2 |  |  | DunedinPoAm |  |  | DunedinPACE |  |
| --- | --- | --- | --- | --- | --- | --- | --- | --- | --- | --- | --- | --- | --- | --- | --- |
|  | ATE | 95% CI | p | ATE | 95% CI | p | ATE | 95% CI | p | ATE | 95% CI | p | ATE | 95% CI | p |
| Social isolation | 0.53 | [-0.39, 1.45] | 0.262 | 0.62 | [0.12, 1.12] | 0.015 | 0.79 | [0.26, 1.33] | 0.004 | 0.014 | [0.004, 0.024] | 0.007 | 0.021 | [-0.001, 0.043] | 0.057 |
| Loneliness | -0.02 | [-0.67, 0.62] | 0.943 | 0.18 | [-0.15, 0.50] | 0.294 | 0.16 | [-0.20, 0.51] | 0.393 | 0.009 | [0.001, 0.016] | 0.019 | 0.014 | [0.001, 0.027] | 0.030 |
| Low positive quality | -0.23 | [-0.86, 0.41] | 0.484 | 0.01 | [-0.32, 0.33] | 0.967 | 0.04 | [-0.32, 0.40] | 0.829 | 0.001 | [-0.006, 0.008] | 0.833 | 0.005 | [-0.008, 0.017] | 0.473 |
| High negative quality | 0.53 | [-0.23, 1.30] | 0.168 | 0.10 | [-0.28, 0.48] | 0.598 | 0.18 | [-0.23, 0.59] | 0.389 | 0.004 | [-0.004, 0.013] | 0.319 | 0.015 | [0.001, 0.029] | 0.039 |

Note: Models were fitted separately for different social connection measures. Each treatment-assignment model adjusted for gender, age, age square, ethnicity, education, employment status, household income, diagnosed psychiatric condition, diagnosed physical health condition, smoking status, baseline wave. The outcome models additionally adjusted for cell type variables.

# Table S3. Results from doubly robust estimation models: sensitivity analysis excluding health-related covariates (N=2343)

|  |  | PhenoAge |  |  | GrimAge |  |  | GrimAge2 |  |  | DunedinPoAm |  |  | DunedinPACE |  |
| --- | --- | --- | --- | --- | --- | --- | --- | --- | --- | --- | --- | --- | --- | --- | --- |
|  | ATE | 95% CI | p | ATE | 95% CI | p | ATE | 95% CI | p | ATE | 95% CI | p | ATE | 95% CI | p |
| Social isolation | 0.57 | [-0.38, 1.51] | 0.240 | 1.15 | [0.56, 1.75] | <0.001 | 1.29 | [0.67, 1.9] | <0.001 | 0.021 | [0.010, 0.032] | <0.001 | 0.025 | [0.006, 0.044] | 0.009 |
| Loneliness | 0.001 | [-0.65, 0.65] | 0.997 | 0.31 | [-0.07, 0.70] | 0.106 | 0.32 | [-0.09, 0.73] | 0.128 | 0.011 | [0.004, 0.019] | 0.004 | 0.018 | [0.005, 0.031] | 0.006 |
| Low positive quality | -0.11 | [-0.75, 0.53] | 0.735 | 0.25 | [-0.13, 0.62] | 0.197 | 0.29 | [-0.11, 0.70] | 0.157 | 0.004 | [-0.003, 0.012] | 0.257 | 0.009 | [-0.004, 0.022] | 0.155 |
| High negative quality | 0.59 | [-0.13, 1.30] | 0.109 | 0.28 | [-0.14, 0.71] | 0.194 | 0.38 | [-0.08, 0.84] | 0.102 | 0.008 | [-0.001, 0.017] | 0.091 | 0.020 | [0.005, 0.035] | 0.009 |

Note: Models were fitted separately for different social connection measures. Each treatment-assignment model adjusted for gender, age, age square, ethnicity, education, employment status, household income, baseline wave. The outcome models additionally adjusted for cell type variables.

# Tables S4. Results from SLCMA models on DunedinPoAm and DunedinPACE: social isolation and loneliness (N=2313†)

| Exposure model | Variables selected | Coefficient [CI] | Partial R^2^ | Total R^2^ |
| --- | --- | --- | --- | --- |
| DunedinPoAm: |  |  |  |  |
| Social isolation | Accumulation | 0.005 [0.002, 0.009] | 0.004 | 0.292 |
| Loneliness | Early exposure (2006/08) | 0.010 [0.003, 0.017] | 0.003 | 0.292 |
| DunedinPACE: |  |  |  |  |
| Social isolation | Accumulation | 0.007 [0.001, 0.013] | 0.002 | 0.209 |
| Loneliness | Middle exposure (2010/12) | 0.021 [0.009, 0.034] | 0.005 | 0.211 |

Note: † These models removed outliers defined by 3 SD of DunedinPoAm/ DunedinPACE as SCLMA is sensitive to outliers. All models adjusted for gender, age, age square, ethnicity, education, employment status, household income, diagnosed psychiatric condition, diagnosed physical health condition, smoking status, baseline wave and cell type variables

# Tables S5. Results from SLCMA models on DunedinPoAm and DunedinPACE sensitivity analysis excluding health-related covariates: social isolation and loneliness (N=2313†)

| Exposure model | Variables selected | Coefficient [CI] | Partial R^2^ | Total R^2^ |
| --- | --- | --- | --- | --- |
| DunedinPoAm: |  |  |  |  |
| Social isolation | Accumulation | 0.008 [0.004, 0.012] | 0.008 | 0.193 |
| Loneliness | Early exposure (2006/08) | 0.012 [0.004, 0.019] | 0.004 | 0.190 |
| DunedinPACE: |  |  |  |  |
| Social isolation | Accumulation | 0.009 [0.003, 0.015] | 0.004 | 0.168 |
| Loneliness | Middle exposure (2010/12) | 0.026 [0.014, 0.039] | 0.007 | 0.171 |

Note: † These models removed outliers defined by 3 SD of DunedinPoAm/ DunedinPACE as SCLMA is sensitive to outliers. All models adjusted for gender, age, age square, ethnicity, education, employment status, household income, baseline wave and cell type variables

LBQ 2006/2008

(n=14768)

Epigenetic clocks 2016

(n=4018)

Merged

(n=2434)

Not merged with clocks data (n=12334, 83.5%)

Not merged with LBQ

(n=1584, 39.4%)

Analytical sample

(n=2343)

Missing data

(n=91, 3.7%)

# Figure S1. Sample selection diagram

# Figure S2. Elbow plots of SLCMA models testing the association of DunedinPoAm and DunedinPACE with social isolation and loneliness: sensitivity analysis excluding health-related covariates
